# Positioning of Chinese time nouns and adverbs: Evidence from corpus, acceptability, and processing studies

**DOI:** 10.1371/journal.pone.0329271

**Published:** 2025-07-30

**Authors:** Jia Yi Chen, Ying Su, Katsuo Tamaoka

**Affiliations:** 1 School of Foreign Languages, Shanghai University, Shanghai, China; 2 Graduate School of Humanities, Nagoya University, Nagoya, Aichi, Japan; Father Muller Charitable Institutions, INDIA

## Abstract

This study examines the syntactic placement and cognitive processing of time nouns and time adverbs in Mandarin Chinese, a language without overt tense morphology, highlighting how these temporal expressions interface with Chinese grammar. Study 1 analyzed a large-scale natural language corpus (BLCU Chinese Corpus) to determine the typical positions of time nouns and time adverbs relative to the subject. The results revealed distinct distributional patterns: time nouns occurred flexibly either before or after the subject, while time adverbs appeared predominantly in post-subject (sentence-internal) positions. Study 2 investigated native Mandarin speakers’ acceptability judgments of sentences with time expressions in various positions. Sentences in which time nouns followed the subject were rated as more acceptable and supported the canonical word order, whereas pre-subject time nouns were acceptable mainly in topicalized contexts. In contrast, time adverbs were strongly preferred in post-subject positions, with only a few exceptions where certain adverbs could be fronted. Study 3 examined the real-time comprehension of these structures using reaction time and accuracy. Results showed that sentences with time expressions in non-canonical positions incurred greater processing costs, while canonical post-subject placements facilitated faster and more accurate processing. These findings suggest that the human sentence processor is sensitive to structural preferences for temporal expressions, mirroring patterns in natural use and grammatical acceptability. By integrating corpus analysis, acceptability judgments, and psycholinguistic data, this study provides a comprehensive account of how time nouns and time adverbs are positioned and processed in Chinese, offering broader implications for understanding temporal reference in tenseless languages.

## Introduction

Time plays a crucial role in specifying when an event described in a sentence takes place. In many European languages, temporal information is conveyed through verbal inflections. For instance, in English, adding the suffix *-ed* to a verb (e.g., *looked*) signals the past tense. Similarly, in Japanese, which is spoken in geographic proximity to China, the past tense is marked by the suffix *-ta*, as in *mita* (‘looked’). In contrast, Mandarin Chinese does not encode tense morphologically, as it lacks inflectional markers that directly indicate temporal reference on the verb [1–3]. Instead, temporal interpretation is achieved through time expressions such as 昨天 (*zuótiān* ‘yesterday’), aspectual markers, and contextual cues [4–5]. While there is ongoing debate as to whether Chinese should be classified as a fully tenseless language [6–7], our use of the term ‘tenseless’ reflects a descriptive focus on the absence of grammatical tense morphology, rather than a strong typological classification. We adopt this characterization to better clarify how time expressions function syntactically in Mandarin in the absence of overt tense marking. In many European languages, although time expressions can be used, they are not required for tense interpretation because tense is already encoded morphologically within the verb. In such languages, these expressions are typically analyzed as time adverbs that modify already tensed predicates. By contrast, in Mandarin Chinese, time expressions function as essential temporal anchors and are more appropriately categorized as time nouns, rather than mere adverbial modifiers. This syntactic and functional distinction plays a key role in how Mandarin speakers interpret temporal reference in the absence of tense morphology.

From a word-order perspective, time nouns in Chinese appear to occupy a position analogous to the specifier of the Tense Phrase ([Spec, TP] or [Spec, T’]) observed in other major languages [8–14]. For example, in the sentence “他昨天打破一个花瓶” (*Tā zuótiān dǎpò yí gè huāpíng*, ‘He broke a vase yesterday’), the time noun 昨天 (*zuótiān*, ‘yesterday’) clearly situates the event in the past, making additional tense marking unnecessary. This “[Spec, TP]-like” position, paralleling tense-related positions in morphologically tense-marking languages, serves as the default placement for time expressions in Chinese. Importantly, Chinese time nouns exhibit positional flexibility: they can appear either before or after the subject [15–21]. Several Chinese linguists [15,22,23] have argued that time nouns are typically placed after the subject, but may be fronted when serving a topicalizing function. According to this topicalization hypothesis, the post-subject position represents the base position for time nouns, while the pre-subject position arises through discourse-driven topicalization.

In addition to time nouns, Chinese also includes a class of temporal expressions known as time adverbs, such as 已经 (*yǐjīng*, ‘already’). Unlike time nouns, time adverbs are generally not found before the subject [24–27] and are not typically topicalized. This suggests a syntactic distinction between time nouns and time adverbs, likely rooted in their differing lexical categories and syntactic behaviors. As a result, time expressions in Chinese are generally divided into two categories: time nouns and time adverbs, each exhibiting distinct syntactic distributions and constraints [28–36].

While prior linguistic literature has discussed the syntactic flexibility of time expressions in Chinese, particularly their ability to appear either before or after the subject, these discussions have largely remained theoretical or based on introspective examples, lacking systematic empirical validation. This study addresses that gap by integrating three complementary sources of evidence: (1) corpus analysis of natural spoken data to uncover objective distributional patterns, (2) acceptability judgment data to assess native speaker intuitions, and (3) sentence processing experiments to examine cognitive preferences during real-time comprehension. By these three methods, the study offers a comprehensive investigation of the positional behavior of time expressions in Chinese, revealing not only their syntactic variability but also potential processing preferences. Moreover, by distinguishing between time nouns and time adverbs, we uncover systematic differences in their positional flexibility and shared tendencies in anchoring event time. Through this multi-method approach, the study contributes empirical evidence to our understanding of how time expressions function in Chinese, bridging theoretical claims with actual language use and processing.

## Background

### Position of time nouns in a sentence

Time nouns in Chinese can be broadly divided into two types: point-in-time nouns and duration nouns [30–32,35,36]. Point-in-time nouns refer to a specific moment when something happens, marking a single point on the timeline. They usually answer the question *“When?”* Examples include 昨天 (*zuótiān*, ‘yesterday’), 去年 (*qùnián*, ‘last year’), and 2000年 (*èr líng líng líng nián*, ‘the year 2000’). Duration nouns, on the other hand, describe how long something lasts. They answer the question *“For how long?”* Examples include 三天 (*sān tiān*, ‘three days’), 一个月 (*yí gè yuè*, ‘one month’), and 一段时间 (*yí duàn shíjiān*, ‘a period of time’). Many of these time nouns have been discussed in earlier studies, including those by Hu [30], Huang & Liao [31], Liu et al. [32], Zhang [35], and Zhao [36].

*Point-in-time* nouns:

**Table:** 

春天	*chūntiān* ‘spring’	秋天	*qiūtiān* ‘autumn’
冬天	*dōngtiān* ‘winter’	去年	*qùnián* ‘last year’
2000年	*èrlínglínglíngnián* ‘the year 2000’	十二点	*shí'èr diǎn* ‘twelve o’clock’
21世纪	*èrshíyī shìjì* ‘21st century’	夏天	*xiàtiān* ‘summer’
今年	*jīnnián* ‘this year’	星期一	*xīngqī yī* ‘Monday’
今天	*jīntiān* ‘today’	一月	*yīyuè* ‘January’
明年	*míngnián* ‘next year’	昨天	*zuótiān* ‘yesterday’

*Duration* nouns:

**Table:** 

半分钟 *bàn fēnzhōng* ‘half a minute’	一个世纪 *yí gè shìjì* ‘one century’
半小时 *bàn xiǎoshí* ‘half an hour’	一个星期 *yí gè xīngqī* ‘one week’
三年 *sān nián* ‘three years’	一秒 *yì miǎo* ‘one second’
十个月 *shí gè yuè* ‘ten months’	一天 *yì tiān* ‘one day’
一百年 *yì bǎi nián* ‘one hundred years’	一周 *yì zhōu* ‘one week’

In Chinese sentences, a time noun can serve various linguistic roles, making its placement flexible. Previous studies on Chinese time nouns [15–21] have suggested that time nouns are generally used after the subject and before the verb.

In English, tense is marked on the verb, which typically appears after the subject. For example, in the sentence ‘We watched the news last night,’ the suffix *-ed* places the event in the past, while the time adverb ‘last night’ specifies the exact time. The tense marker *-ed* does not specify when the event occurred but is consistent with the time indicated by the adverb. Considering this sentence as [_TP_ NP(we) [_T’_ T(-*ed* PST) [_VP_ V(watch) NP(the news)]]]. The past tense is placed the specifier of noun place as [Spec, TP] [8–14]. In this structure, time is indicated after the subject. In Chinese, the time noun ‘last night,’ expressed as 昨天晚上 (*zuótiān* ‘yesterday’ + *wǎnshàng* ‘evening’), also appears after the subject, as in 我们昨天晚上看了新闻 (*wǒmen zuótiān wǎnshàng kàn le xīnwén,* ‘We watched the news last night’). By noting that Chinese lacks overt tense morphology, there remains a notable similarity in word order: Chinese time nouns occupy a slot parallel to the tense position in English ([Spec, TP]).

Native Chinese speakers often place a time noun before the subject. Among Chinese linguists [15,22,23], this order is interpreted as topicalization. Chao [15], Li & Thompson [22], and Xu & Langendoen [23] consider Chinese as one of the languages with a topic-comment structure. However, since Chinese has no topic marker, there is no definite syntactic distinction between scrambling and topicalization, which can result in the word order where the time noun is fronted. In this paper, we refer to this order as a topicalized order based on the studies of Chinese linguists [15,22,23]. Due to this topicalized order, a time noun creates two distinct word orders, as shown in Sentence (1) and Sentence (2). In these orders, NP refers to a noun phrase, TN refers to a time noun, V refers to a verb, *-sub* refers to the subject, and *-obj* refers to the object.

(1) Basic word order: Subject + Time Noun + Verb + Object

**Table:** 

我们	明天	去	学校。
*Wǒmen*	*míngtiān*	*qù*	*xuéxiào.*
NP*-sub*(We)	TN(tomorrow)	V(go)	NP*-obj*(school)

“We will go to school tomorrow.”

(2) Topicalized word order: Time Noun + Subject + Verb + Object

**Table:** 

明天	我们	去	学校。
*Míngtiān*	*wǒmen*	*qù*	*xuéxiào.*
TN(tomorrow)	NP*-sub*(We)	V(go)	NP*-obj*(school)

In Sentence (1), the subject 我们 (*wǒmen*) is at the beginning of the sentence, following the time noun, creating the basic word order, “[Spec, TP]-like” position in Chinese. In Sentence (2), 明天 (*míngtiān*) is topicalized at the beginning of the sentence, emphasizing the time ‘we will go to school’ and thereby highlighting that the action will occur tomorrow rather than at another time. Thus, the time noun is placed at the beginning of the sentence to focus on the temporal context. Consequently, two word orders are created by positioning the time nouns either before or after the subject.

### Position of time adverbs in a sentence

Time adverbs in Chinese generally do not precede the subject [24,26,27]. Based on a corpus study using the Modern Chinese Corpus developed by the Research Center for Chinese Linguistics at Peking University, Yang [25] analyzed the frequency and syntactic position of 62 time adverbs. The findings show that the regular position for time adverbs is after the subject, whereas the position before the subject is considered non-regular. This preference can be explained by the syntactic role of time adverbs in Chinese. Most time adverbs modify the verb phrase and are therefore typically placed after the subject within the main part of the sentence. However, a smaller group of time adverbs functions at the sentence level, modifying the entire proposition rather than just the action. These sentence-level adverbs contribute to the overall temporal framing and are more flexible in their placement, sometimes appearing before the subject. This distinction in function explains why some time adverbs can only appear after the subject, while others can also appear before it. Notably, such flexibility is often associated with disyllabic or longer adverbs [25,26,37–39]. For example, disyllabic adverbs such as 最近 (*zuìjìn*, ‘recently’) may occur either before or after the subject, whereas time adverbs consisting of a single syllable or a single hanzi (e.g., 老 *lǎo* ‘always’) only appear after the subject.

In this study, disyllabic (two-syllable) time adverbs are investigated to identify their positions in a sentence. The major time adverbs considered in the present study are listed as follows:

**Table:** 

曾经 *céngjīng* ‘once’	已经 *yǐjīng* ‘already’
迟早 *chízǎo* ‘sooner or later’	一同 *yìtóng* ‘together’
忽然 *hūrán* ‘suddenly’	一直 *yìzhí* ‘continuously’
就要 *jiùyào* ‘about to’	预先 *yùxiān* ‘in advance’
马上 *mǎshàng* ‘immediately’	暂且 *zànqiě* ‘for the time being’
仍然 *réngrán* ‘still’	正在 *zhèngzài* ‘in the process of’
随后 *suíhòu* ‘afterwards’	逐步 *zhúbù* ‘gradually’
向来 *xiànglái* ‘always’	总是 *zǒngshì* ‘always’

A disyllabic time adverb creates two distinct word orders, as shown in Sentence (3) and Sentence (4). In these sentences, NP refers to a noun phrase, TA refers to a time adverb, V refers to a verb, *-sub* refers to the subject, and *-obj* refers to the object.

(3) Basic word order: Subject + Time Adverb + Verb + Object

**Table:** 

我	早晚	会解释	这件事情的。
*Wǒ*	*zǎowǎn*	*huì jiěshì*	*zhè jiàn shìqíng de .*
NP*-sub*(I)	TA(Sooner or later)	V(explain)	NP*-obj*(thing)

“Sooner or later, I will explain this whole thing.”

(4) Topicalized word order: Time Adverb + Subject + Verb + Object

**Table:** 

早晚	我	会解释	这件事情的。
*Zǎowǎn*	*wǒ*	*huì jiěshì*	*zhè jiàn shìqíng de .*
TA(Sooner or later)	NP*-sub*(I)	V(explain)	NP*-obj*(thing)

Although Sentences (3) and (4) have roughly the same meaning, they differ in semantic scope. In Sentence (3), the time adverb 早晚 (*zǎowǎn* ‘sooner or later’) positioned after the subject has narrower scope and qualifies the time when the action takes place. This is considered the basic word order in Chinese [24–27]. By contrast, in Sentence (4), the time adverb 早晚 appears at the beginning of the sentence. This sentence has a wider scope and can qualify the whole event, emphasizing the time when ‘I explained the matter.’

However, not all disyllabic time adverbs can be positioned before the subject. Some disyllabic time adverbs seem to be restricted in their semantic expression [26,37,39,40]. This restriction is exemplified in Sentences (5) and (6) with the time adverb 已经 (*yǐjīng*, ‘already’). The time adverb 已经 cannot be placed at the beginning of the sentence before the subject, making Sentence (6) incorrect, as indicated by the asterisk (*). In these sentences, PP refers to a prepositional phrase. -ASP refers to aspect.

(5) Subject + Time Adverb + Prepositional Parse + Verb

**Table:** 

他们	已经	高中	毕业了。
*Tāmen*	*yǐjīng*	*gāozhōng*	*bìyè le.*
NP*-sub*(they)	TA(already)	PP(from high school)	V(graduate)-ASP

‘They have already graduated from high school’.

(6) Time Adverb + Subject +Prepositional Parse + Verb

**Table:** 

*已经	他们	高中	毕业了。
*Yǐjīng*	*tāmen*	*gāozhōng*	*bìyè le.*
TA(already)	NP*-sub*(they)	PP(from high school)	V(graduate)-ASP

The position of time adverbs in a sentence varies depending on the specific adverb. Previous studies have noted that most time adverbs tend to occur after the subject, while certain disyllabic forms (e.g., 最近 ‘recently’) may also appear in the pre-subject position [25–26,37–39]. These observations raise important questions about how time adverbs compare with time nouns in terms of syntactic distribution, and what factors may influence their placement relative to the subject. To address these questions, the present study steadily investigates the sentential positions of time nouns and time adverbs in Mandarin Chinese, with a particular focus on their pre- and post-subject placement. We adopt a three-methodological approach to examine this issue from complementary perspectives: a corpus analysis to provide objective evidence from natural language use, an acceptability judgment study to capture native speakers’ intuitive preferences, and a sentence processing experiment to reveal real-time cognitive behavior.

## Study 1 – Corpus study of time nouns and time adverbs

Study 1 conducted a corpus analysis to explore the distributional tendencies of time nouns and time adverbs in natural discourse. Since conversational corpus closely reflect the authentic use of Chinese, this study utilized the spoken Chinese corpus from the BLCU Chinese Corpus (BCC) [41]. By examining their frequencies of occurrence before and after the subject, Study 1 investigated whether both time nouns and time adverbs preferentially occupy the “[Spec, TP]-like” position following the subject.

## Materials and methods

### Selection of time nouns and time adverbs

The time nouns were selected based on the *Chinese Proficiency Grading Standards for International Chinese Language Education* [42] issued by the Center for Language Education and Cooperation under the Ministry of Education, China. These standards provide guidelines for standardizing the vocabulary used in Chinese proficiency testing. Meanwhile, the time adverbs were selected in accordance with the classification system proposed by Xu and Wu [43] in their study, *Experimental Study on the Classification System of Time Adverbs in Modern Chinese.*

### Corpus and search methods

Study 1 utilized the conversational corpus from the BCC to better capture natural Chinese language usage. This corpus includes conversational data from platforms such as Weibo and movie subtitles. For the frequency analysis, 30 target time expressions (15 time nouns and 15 time adverbs) were extracted from the corpus. To ensure the dataset’s representativeness, 500 sentences were collected for each time expression, focusing specifically on those containing explicit subjects. This approach allowed us to determine whether each time noun or time adverb appeared before or after the subject. Because the BCC imposes a download limit of 10,000 records per query, time expressions with more than 10,000 occurrences were sampled from the first 10,000 entries at intervals of every 20 occurrences, yielding 500 selections. For example, the expression 昨天 (*zuótiān*, ‘yesterday’) appears 165,052 times in the corpus, but only 10,000 records are downloadable. From those 10,000 records, 500 sentences were selected by taking every 20th occurrence, of which 218 contained explicit subjects. These 218 sentences were then used to calculate the frequency of ‘昨天’ appearing before and after the subject.

For time expressions with fewer than 10,000 occurrences, sentences were proportionally sampled to obtain a set of 500 sentences by adjusting the selection interval. For example, 将来 (*jiānglái*, ‘future’) appears 6,479 times, and proportional sampling ensured a consistent dataset of 500 sentences. When the total number of available sentences was less than 500, all existing sentences were included. Consequently, expressions such as 前天 (*qiántiān*, ‘the day before yesterday’) with 498 sentences, 预先 (*yùxiān*, ‘in advance’) with 129 sentences, and 逐步 (*zhúbù*, ‘gradually’) with 298 sentences were included in their entirety. After filtering, Table 1 presented the retrieved time expressions and their corresponding frequencies of use in Study 1.

**Table 1:** Positional frequencies of time expressions in the conversational corpus.

Time Expression	Pinin	Meaning	Total Frequencies	Frequencies	
				Before Subject	After Subject
昨天	*zuótiān*	yesterday	165,052	63	155
今天	*jīntiān*	today	538,830	46	143
明天	*míngtiān*	tomorrow	336,374	30	120
前天	*qiántiān*	the day before yesterday	13,519	45	131
后天	*hòutiān*	the day after tomorrow	33,473	21	99
早上	*zǎoshàng*	morning	117,134	16	66
中午	*zhōngwǔ*	noon	68,478	10	58
下午	*xiàwǔ*	afternoon	127,867	22	93
晚上	*wǎnshàng*	evening	269,009	7	86
目前	*mùqián*	currently	22,148	97	69
现在	*xiànzài*	now	770,934	60	152
过去	*guòqù*	the past	96,765	0	0
将来	*jiānglái*	the future	6,479	83	79
去年	*qùnián*	last year	40,194	55	115
今年	*jīnnián*	this year	82,251	12	146
		Total		567	1,512
	Chi-square test of goodness-of-fit			*χ*^*2*^(1) = 429.545, *p* < .001	
	Independent-samples *t*-test			*t*(28) = −2.227, *p* < .05	
已经	*yǐjīng*	already	376,788	0	303
预先	*yùxiān*	beforehand	129	0	20
随后	*suíhòu*	subsequently	676	34	319
逐步	*zhúbù*	gradually	298	0	161
马上	*mǎshàng*	immediately	63,667	6	152
忽然	*hūrán*	suddenly	6,241	8	101
仍然	*réngrán*	still	2,498	1	239
一直	*yìzhí*	continuously	293,623	0	258
总是	*zǒngshì*	always	56,534	11	290
暂且	*zànqiě*	temporarily	774	1	203
就要	*jiùyào*	about to	65,420	0	85
正在	*zhèngzài*	in the process of	37,797	0	221
一同	*yìtóng*	together	1,786	0	66
迟早	*chízǎo*	sooner or later	5,023	16	161
向来	*xiànglái*	always	2,065	13	317
		Total		90	2,896
	Chi-square test of goodness-of-fit			*χ*^*2*^(1) = 2,636.851, *p* < .001	
	Independent-samples *t*-test			*t*(28) = −9.417, *p* < .001	

## Results

To identify the more frequent position of time expressions (pre-/post- subject), we first performed a chi-square goodness-of-fit test on the total frequencies of time nouns and time adverbs. Time nouns appeared 2,079 times in total, with 567 instances (27%) occurring before the subject and 1,512 instances (73%) occurring after the subject. The chi-square analysis revealed a significant preference for positions after the subject, *χ²*(1) = 429.545, *p* < .001. Similarly, time adverbs were observed 2,986 times overall, with 90 instances (3%) positioned before the subject and 2,896 instances (97%) positioned after. This analysis also demonstrated a strong bias toward positions after the subject, *χ²*(1) = 2,636.851, *p* < .001.

To further quantify the positional differences, we performed an independent-samples *t*-test on the 15 time nouns, after transforming the frequencies to a natural log by *log*_*e*_(X + 0.5) [44]. Using this method, the frequency of 0 becomes −0.69 by *log*_*e*_(0 + 0.5). The result of *t*-test showed a significant difference between before and after the subject, *t*(28) = −2.227, *p* < .05, showing time nouns occurring more frequently after the subject. Likewise, the 15 time adverbs showed the same trend, *t*(28) = −9.417, *p* < .001, indica*t*ing that they occurred exclusively after the subject. Thus, the corpus study of Study 1 indicated that both time nouns and time adverbs preferentially occupy the “[Spec, TP]-like” position following the subject.

## Discussion

Study 1 employed a corpus analysis to investigate the distributional tendencies of time nouns and time adverbs in natural discourse. By examining their frequencies of occurrence before and after the subject in a conversational corpus, the study aimed to determine whether these time expressions preferentially occupy the “[Spec, TP]-like” position following the subject. A chi-square goodness-of-fit test revealed a significant deviation from a uniform distribution, suggesting that the post-subject position (“[Spec, TP]-like” position) serves as the default placement for time expressions in spoken Chinese. This result was further corroborated by an independent-samples *t*-test, which showed that both time nouns and time adverbs strongly favored the position after the subject, with time adverbs displaying an even greater inclination. These findings are further explored in Study 2, which examines native Chinese speakers’ subjective perceptions of time nouns and time adverbs placed in different syntactic positions.

## Study 2 – Acceptability for time nouns and time adverbs

Building on the findings of Study 1 examined the positional tendencies of time nouns and time adverbs in natural discourse. Study 1 demonstrated that both time nouns and time adverbs strongly favor the post-subject position in conversational contexts, corpus data alone cannot confirm how native speakers judge the grammatical acceptability of pre-subject positions. Thus, Study 2 evaluated the acceptability ratings of these time expressions in different syntactic positions.

## Materials and methods

### Participants

Thirty native Chinese-speaking university students (15 females and 15 males) at Shanghai University participated in an acceptability judgment task. Their ages ranged from 19 to 29 years, with a mean age of 23 years and 6 months (*SD* = 2 years and 11 months). Ethical approval for this study was obtained from the Shanghai University Research Ethics Committee. All participants provided written informed consent and received monetary compensation for their participation. To protect privacy, all data were securely stored, and numerical pseudonyms were used to anonymize participant identities.

### Stimulus sentences

In Study 2, the acceptability judgment task included sentences containing 15 time nouns (e.g., 昨天 *zuótiān* ‘yesterday’, 今天 *jīntiān* ‘today’) and sentences containing 15 time adverbs (e.g., 已经 *yǐjīng* ‘already’, 目前 *mùqián* ‘currently’). The time expression used in Study 2 was the same as in Study 1. Each time noun or time adverb was placed before and after the subject, resulting in two different sentence orders: ‘Time Noun or Time Adverb (T) + Subject (S) + Verb (V)-aspect + Object (O)’ and ‘Subject (S) + Time Noun or Time Adverb (T) + Verb (V)-aspect + Object (O).’ This procedure resulted in a total of 60 sentences, comprising 15 targets × 2 types of time expressions (time nouns and time adverbs) × 2 positions (pre-/post- subject). For time nouns, Sentence (7) appeared before the subject, and Sentence (8) appeared after the subject. For time adverbs, Sentence (9) appeared before the subject, and Sentence (10) appeared after the subject. All stimulus sentences used in Study 2 are provided in S1 Appendix.

(7) TN (Time Noun) + S (Subject) + V (Verb)-aspect + O (Object)

**Table:** 

昨天	我	去了	超市。
*Zuótiān*	*wǒ*	*qù le*	*chāoshì.*
TN(yesterday)	NP-sub(I)	V(go)-ASP	NP-obj(supermarket)

‘I went to the supermarket yesterday.’

(8) S (Subject) + TN (Time Noun) + V (Verb)-aspect + O (Object)

**Table:** 

我	昨天	去了	超市。
*Wǒ*	*zuótiān*	*qù le*	*chāoshì.*
NP-sub(I)	TN(yesterday)	V(go)-ASP	NP-obj(supermarket)

(9) TA (Time Adverb) + S (Subject) + V (Verb)-aspect + O (Object)

**Table:** 

已经	鹏鹏	完成了	这项任务。
*Yǐjīng*	*péngpeng*	*wánchéng le*	*zhè xiàng rènwù.*
TA(already)	NP-sub(Pengpeng)	V(accomplish)-ASP	NP-obj(task)

‘Peng Peng has accomplished this task.’

(10) S (Subject) + TA (Time Adverb) + V (Verb)-aspect + O (Object)

**Table:** 

鹏鹏	已经	完成了	这项任务。
*Péngpeng*	*yǐ jīng*	*wánchéng le*	*zhè xiàng rènwù.*
NP-sub(Pengpeng)	TA(already)	V(accomplish)-ASP	NP-obj(task)

All sentences were constructed as minimal pairs that differed only in the position of the temporal expression (pre-subject vs. post-subject). Sentence structure and lexical content were carefully controlled to ensure uniformity in length and complexity. An independent-samples *t*-test confirmed that sentence length did not significantly differ between the time noun and time adverb conditions, *t*(28) = 0.41, *p* = .686, *ns*. Moreover, all lexical items were selected from high-frequency modern Chinese vocabulary, and all participants were native speakers of Mandarin Chinese, making lexical complexity unlikely to have influenced their judgments.

### Readability assessment

Prior to the main experiment, a separate readability rating task was conducted to ensure the suitability of the stimulus sentences. Twenty-six native Mandarin speakers (14 females, 12 males; age range: 19 years 3 months to 39 years 4 months; *M* = 24 years 3 months, *SD* = 3 years 10 months), who did not participate in the main experiment, evaluated the readability of all 60 sentences on a 5-point Likert scale (1 = very difficult to read, 5 = very easy to read). All four conditions of time noun pre-/post-subject time noun and pre-/post-subject time adverb received mean ratings above 4.30, indicating high readability. The specific mean scores were as follows: Time noun–subject–verb–object (TN + S + VO): *M* = 4.97, *SD* = 0.21; Subject–time noun–verb–object (S + TN + VO): *M* = 4.99, *SD* = 0.10; Time adverb–subject–verb–object (TA + S + VO): *M* = 4.36, *SD* = 0.92; and Subject–time adverb–verb–object (S + TA + VO): *M* = 4.97, *SD* = 0.20. The internal consistency of the ratings was excellent (Cronbach’s *α* = 0.946). These results confirm that the stimuli used in Study 2 were highly readable and suitable for use in the acceptability judgment task.

### The questionnaire for acceptability judgments

Each participant received a printed questionnaire containing 60 sentences. They rated the naturalness of each sentence using a 7-point Likert scale ranging from −3 (totally unacceptable) to +3 (very acceptable). To minimize proximity effects and reduce the risk of response bias, sentences were presented in a randomized and stratified order, ensuring that minimally different versions of the same base sentence (e.g., differing only in the position of the time expression) did not appear consecutively. Although each base sentence was included twice with different temporal placements, the randomized presentation and offline format encouraged participants to evaluate each sentence independently. This design helped mitigate potential strategy effects and limited participants’ awareness of the study’s specific focus on word order. Furthermore, the acceptability judgment task demonstrated high internal reliability (Cronbach’s *α* = 0.949), indicating consistent responses across items and supporting the robustness of the data.

## Results

### Data for acceptability ratings

A total of 1,800 responses by 30 participants using a −3 to +3 scale for the 60 stimulus sentences were analyzed. This analysis was conducted using a linear mixed-effects (LME) model [45] implemented with the *lme4* package [46] within R [47]. Results from multiple models were compared using the Akaike Information Criterion (AIC, Akaike’s Information Criterion, compared using maximum likelihood estimation) [48].

### Results of LME model analyses

The use of a two-factor (Position × Time Type) linear mixed-effects model was theoretically motivated. In Chinese linguistics, time expressions are typically divided into two distinct types (time nouns and time adverbs) each with different syntactic characteristics and expected positional preferences. To systematically examine both the independent and joint effects of these factors, a 2 × 2 factorial design was appropriate and necessary. The interaction term allowed us to test whether the effect of position differed between time nouns and time adverbs, which is central to our research question.

**Fig 1:**
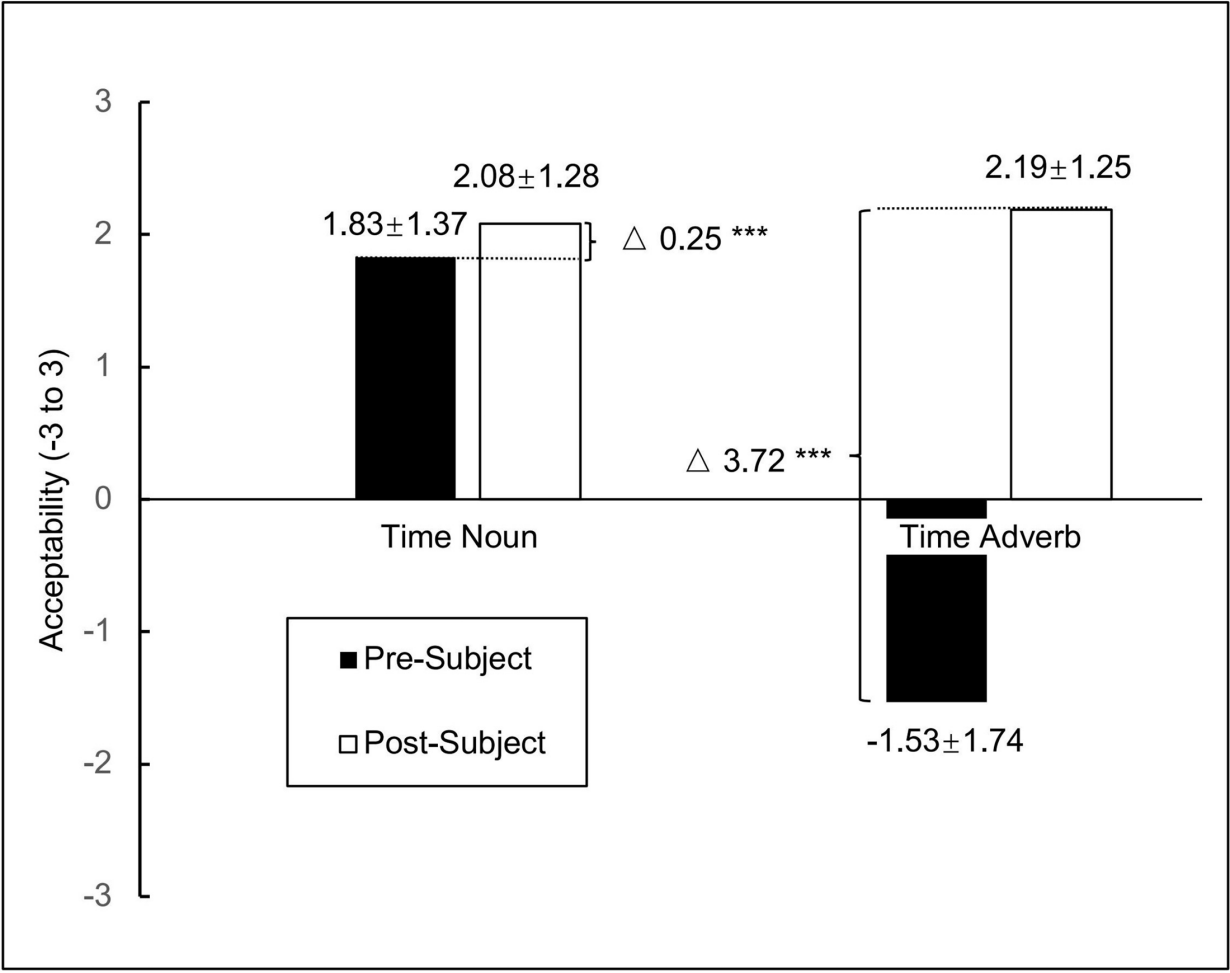
Acceptability ratings for time expressions in different positions. *Note*. *** *p* < .001. The values represent means of acceptability ratings, and the values after ± indicate standard errors. △ represents the difference in acceptability ratings.

Acceptability judgment scores were analyzed using the *lmer* function with restricted maximum likelihood estimation [49]. Satterthwaite’s approximations were applied via the *lmer*Test package to calculate *p*-values for each model [50]. The best-fit LME model was selected based on model comparisons using AIC [48]. The means (*M*) and standard deviations (*SD*) for acceptability judgments of various time expressions in different positions were reported in Table 2 and Fig 1. The results of the LME analysis were presented in Table 3. The two fixed effects were time expressions (time nouns and time adverbs) and time positions (pre-/post-subject), recorded as 1 and −1 for the LME analysis, respectively. The random effects included participants and sentences.

**Table 2:** Descriptive statistics of acceptability ratings for time expressions.

Time Expressions	Time Positions	*M*	*SD*
Time Nouns	Before subject (TN + S + V + O)	1.83	1.37
	After subject (S + TN + V + O)	2.08	1.28
Time Adverbs	Before subject (TA + S + V + O)	−1.53	1.47
	After subject (S + TA + V + O)	2.19	1.25

*Note*. *M* = mean. *SD* = standard deviation.

**Table 3:** LME analysis results for time expressions in different positions.

Variables	Estimate	*SE*	*df*	*t* value	Pr(>|t|)	*p*
(Intercept)	4.833	0.180	59.58	26.838	*p* < .001	^***^
Time expression	−3.367	0.195	34.05	−17.310	*p* < .001	^***^
Position	0.247	0.084	1739.00	2.937	*p* < .01	^**^
Time expression ^*^Position	3.476	0.119	1739.00	29.258	*p* < .001	^***^

*Note*. Subjects = 30. Item = 30. Position (before and after the subject) =2. Time expression (time noun and time adverb) = 2. Total Observation = 1,800. *SE* = standard error. **df* *= degree of freedom.

** *p* < .01.

*** *p* < .001.

As illustrated in Table 3, a significant interaction between time expression type and position was observed, *t*(1739) = 29.258, *p* < .001. This interaction suggests that native Chinese speakers’ acceptability judgments varied depending on both the type of temporal expression and its position in the sentence. Specifically, time nouns were rated as highly acceptable both before the subject (*M* = 1.83, *SD* = 1.37) and after the subject (*M* = 2.08, *SD* = 1.28), indicating a high degree of positional flexibility. In contrast, time adverbs showed a strong positional asymmetry: ratings were very low when they appeared before the subject (*M* = −1.53, *SD* = 1.47), but significantly higher when placed after the subject (*M* = 2.19, *SD* = 1.25). In addition, a significant main effect of position was found, *t*(1739) = 2.937, *p* < .01, wi*t*h post-subject positions receiving overall higher acceptability ratings (*M* = 2.13, *SD* = 1.27) than pre-subject positions (*M* = 0.15, *SD* = 2.37). The main effect of time expression type was also significant, *t*(34) = −17.310, *p* < .001, indicating that time nouns (*M* = 1.96, *SD* = 1.33) were rated more acceptable overall than time adverbs (*M* = 0.33, *SD* = 2.47).

**Fig 2:**
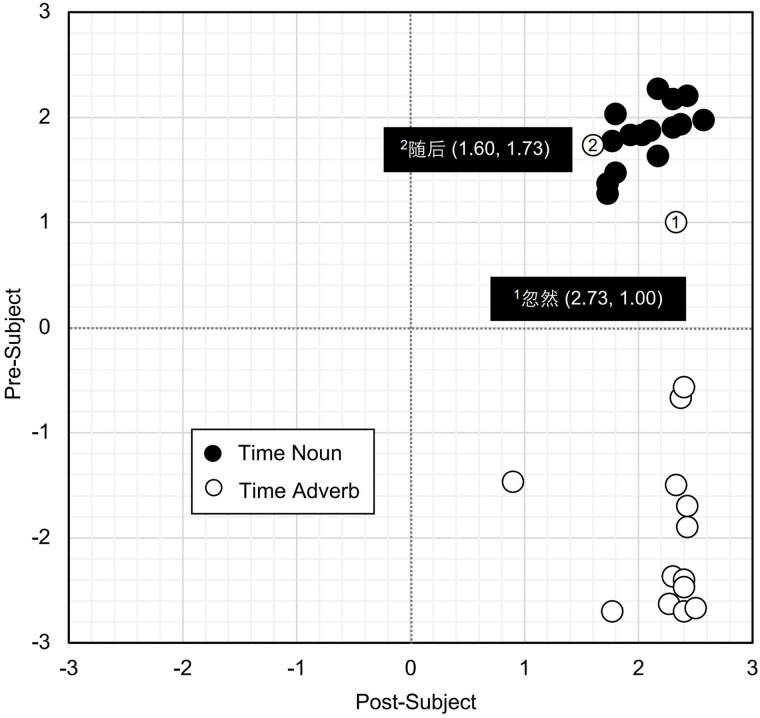
Acceptability ratings for time expressions in pre-/post-subject positions. *Note*. The values for the time adverbs 忽然 and 随后 on the left of the parentheses indicate acceptability scores in the post-subject position, while the values on the right indicate scores in the pre-subject position.

The acceptability ratings of the 15 time nouns and 15 time adverbs used in the stimulus sentences of Study 2 are depicted in Fig 2 for positions both before and after the subject. As shown in Fig 2, the acceptability ratings of all time nouns, whether placed before or after the subject, are clustered at +1 or higher, indicating high acceptability. This clustering clearly indicates that time nouns can appear flexibly before and after the subject. On the other hand, with two exceptions, time adverbs were generally highly acceptable only when positioned after the subject. As seen in Fig 2, two time adverbs functioned similarly to time nouns. As shown in Number 1 of Fig 2, 忽然 *(hūrán*, ‘suddenly’) rated 1.73 after the subject and 1.00 before the subject. Likewise, as shown in Number 2 of Fig 2, 随后 (*suíhòu*, ‘subsequently’) rated 1.60 after the subject and 1.73 before the subject. These two time adverbs appear to be perceived by native Chinese speakers in a similar way to time nouns, whether they are positioned before or after the subject. Therefore, Study 2 indicates that time adverbs are generally more acceptable when positioned after the subject, with the exception of the two mentioned cases.

## Discussion

Time nouns and time adverbs in Chinese appear to be distinguished by their sentence positions. The interaction between the two positions (pre-/post-subject) and the two types of time expressions (time nouns or time adverbs) was significant. All time nouns placed both before and after the subject were highly acceptable. In contrast, time adverbs generally achieved high acceptability only when positioned after the subject [24,26,27], with exceptions in 忽然 (*hūrán*) and 随后 (*suíhòu*). These exceptions patterned similarly to time nouns, suggesting they may function differently from typical time adverbs. Furthermore, as shown in Fig 1, time nouns placed after the subject received higher acceptability ratings than those placed before it. According to previous studies, the basic position of time nouns is likely after the subject (“[Spec, TP]-like” position), while pre-subject placement may indicate topicalization [15,22,23]. Similarly, time adverbs placed before the subject received low acceptability ratings, implying that moving them from their basic position (post-subject) imposes greater syntactic constraints.

## Study 3 – Sentence processing of time nouns and time adverbs

Study 3 examined whether the acceptability patterns identified in Study 2 align with cognitive processing during the comprehension of sentences containing these time expressions before and after the subject. By analyzing reaction times and accuracy rates, Study 3 aims to reveal any differences in sentence processing when time expressions are positioned before versus after the subject.

## Materials and methods

### Participants

Thirty-seven native Chinese-speaking university students (28 females, 9 males) at Shanghai University participated in a sentence correctness decision task. Their ages ranged from 20 to 30 years, with a mean age of 23 years and 5 months (*SD *= 2 years and 4 months). None of them participated in Study 2. Ethical approval for this study was obtained from the Shanghai University Research Ethics Committee. All participants provided written informed consent prior to participation. Each participant received monetary compensation for taking part in the study. To ensure confidentiality, all data were securely stored, and participant identities were anonymized using numerical pseudonyms.

### Stimulus sentences

To examine whether the patterns observed in Study 2 would generalize to a different context and to minimize potential item-specific effects, Study 3 employed a new set of stimulus sentences. In constructing these stimuli, each of the 16 time nouns and 16 time adverbs was placed either before or after the subject, yielding two types of Chinese sentence structures:

1. Time Noun or Time Adverb (T) + Subject (S) + Verb (V)-aspect + Object (O)
2. Subject (S) + Time Noun or Time Adverb (T) + Verb (V)-aspect + Object (O)

This procedure produced a total of 64 correct sentences, consisting of 16 target expressions × 2 types of time expressions (time nouns vs. time adverbs) × 2 positions (pre-/post-subject). All 64 correct stimulus sentences used in Study 3 are provided in S2 Appendix. Because the two positions were counterbalanced, each set of stimulus sentences contained 32 correct items.

To prevent participants from inferring the study’s purpose, 40 additional correct filler sentences were created (20 sentences × 2 positions), which were also counterbalanced. In addition, 52 sentences containing grammatical and/or semantic errors (e.g., 我喝了一条裤子, ‘I drank a pair of pants’) were generated without counterbalancing. As a result, two sets of 104 sentences were compiled, each set comprising 52 correct sentences (32 target + 20 filler) and 52 incorrect sentences. Each of these lists was then distributed among two participant groups.

All sentences in Study 3 were constructed as minimal pairs, differing only in the syntactic position of the temporal expression. Sentence length and lexical content were strictly controlled across all conditions. An independent-samples *t*-test confirmed that sentence length did not significantly differ between the noun and adverb conditions, *t*(28) = 0.50, *p* = .620, *ns*. As all lexical items were drawn from high-frequency modern Chinese vocabulary and all participants were native speakers of Mandarin Chinese, lexical complexity was unlikely to have affected sentence processing.

### Readability assessment

Prior to the main experiment, a separate readability rating task was conducted with 26 native Mandarin speakers (none of whom participated in the main study) to assess the readability of the 64 stimulus sentences. Participants rated each sentence on a 5-point Likert scale (1 = very difficult to read, 5 = very easy to read). The mean readability scores for the four conditions were as follows: Time noun–subject–verb–object (TN + S + VO), *M* = 4.99, *SD* = 0.12; Subject–time noun–verb–object (S + TN + VO), *M* = 4.98, *SD* = 0.17; Time adverb–subject–verb–object (TA + S + VO), *M* = 4.30, *SD* = 0.92; and Subject–time adverb–verb–object (S + TA + VO), *M* = 4.96, *SD* = 0.29. The internal consistency of the ratings was excellent (Cronbach’s *α* = 0.945). These results confirm that the stimuli used in Study 3 were uniformly readable and suitable for use in the processing task.

### Procedure

A Chinese sentence correctness decision task was conducted on 37 native Chinese speakers. An eye fixation symbol (++++++++) was initially presented at the center of the computer screen for 1,000 ms, after which a target sentence replaced it. Participants were then required to decide whether the sentence was a correct Chinese sentence (pressing the YES key for correct and the NO key for incorrect). The next trial appeared after a 500 ms interval. All stimulus sentences were randomly presented to each participant. Participants were instructed to complete the task as quickly and accurately as possible. When no response was made within 10,000 milliseconds, the message “No Response” was displayed and recorded as incorrect. Twelve practice items were provided to each participant before the commencement of the actual experiment.

### Analysis

The accuracy and reaction times data collected from the sentence correctness decision task were analyzed using the linear mixed effect (LME) models [45] and the *lme4* package [46]. The two fixed effects were trial and word order (four sentence conditions). The random effects were participants and stimulus sentences. The data for reaction times consisted only of data from trials with correct judgments. Satterthwaite’s approximations [51] were used via the *lmer*Test package to generate *p*-values for each model [50] using the restricted maximum likelihoods [49].

## Results

### Results of LME model analyses for accuracy data

A total of 1,184 responses (37 participants × 32 semantically and grammatically correct items) were analyzed. The fixed factors were trial and word order. The trial was centralized into *z*-values, coded as “trial.z.” The two random factors were participant and stimulus sentences. According to model comparisons using AIC [48], the final best-fit LME model was *glmer*(acc ~ trial.z + position * type + (1 + position | subject) + (0 + position | item). The means (*M*) and standard deviations (*SD*) for accuracies are presented in Table 4.

**Table 4:** Descriptive statistics for accuracies in Chinese sentences.

Time Expressions	Time Positions	*M*	*SD*
Time Noun	Before subject (TN + S + V + O)	0.90	0.30
	After subject (S + TN + V + O)	0.98	0.15
Time Adverb	Before subject (TA + S + V + O)	0.25	0.43
	After subject (S + TA + V + O)	0.98	0.13

*Note*. *M* = mean. *SD* = standard deviation.

The result of the best-fit LME model is reported in Table 5. Trial order did not significantly affect accuracy, [*z* = −1.467, *ns*], indicating that task performance accuracy did not change as the experiment progressed. The reference for time type and word order was defined as the baseline configuration: time adverbs positioned after the subject. As presented in Table 5, accuracy significantly decreased when time expressions were positioned before the subject [*z* = −6.504, *p* < .001], suggesting that pre-subject positions impose greater processing difficulty. Although the type of time expression (time noun or time adverb) did not independently influence accuracy, [*z* = −0.636, *ns*], the interaction between position and type was significant, [*z* = 5.301, *p* < .001]. This indicates that the positional effect varied depending on the type of time expression: time adverbs were processed significantly less accurately when placed before the subject, whereas time nouns exhibited relatively consistent accuracy regardless of position.

**Table 5:** LME analysis results for accuracies in Chinese sentences.

Variables	Estimate	*SE*	*z* value	Pr(>|z|)	*p*
(Intercept)	5.429	0.943	5.757	*p* < .001	^***^
trial.z	−0.233	0.159	−1.467	*p* = 0.142	*ns*
Position (beforeS)	−8.034	1.235	−6.504	*p* < .001	^***^
Type (time noun)	−0.394	0.619	−0.636	*p* = 0.525	*ns*
Position^*^Type	7.590	1.432	5.301	*p* < .001	^***^

*Note.* Subjects = 37. Item = 32. Type (time noun and time adverb) = 2. Position (before and after the subject) = 2. Total Observation = 1,184. *SE* = standard error.

*** *p* < .001. *ns* = non significant.

When analyzing the positions of time nouns relative to the subject, which used the post-subject position as a reference, it was found that sentences with time nouns placed before the subject were significantly more likely to be perceived as incorrect than those placed after the subject [*z* = 2.804, **p* *< .01]. Similarly, analysis of time adverb positions revealed that sentences with time adverbs placed before the subject were significantly more likely to be perceived as incorrect compared to those placed after the subject [*z* = −3.949, *p* < .001].

### Results of LME model analyses for reaction time data

After removing 264 incorrectly answered items from the 1,184 semantically and grammatically correct items, the remaining 920 correctly answered items were analyzed for reaction times. Based on the Box-Cox power transformation technique [52,53], a logarithmic transformation (natural log) was applied to the reaction times to attenuate any skewness in their distribution. Reaction times were analyzed using the *lmer* function with restricted maximum likelihood [49]. Satterthwaite’s approximations [51] were applied via the *lmerTest* package to generate *p*-values for each model [50]. According to model comparisons using AIC [48], the best-fit LME model was *lmer*((log(rt) ~ trial.z + position*type + (1 + position|subject) + (0 + position|item), timert). The means (*M*) and standard deviations (*SD*) for the 920 responses are presented in Table 6. Based on the best-fit LME model, potentially influential outliers with absolute standardized residuals exceeding 2.5 standard deviations were removed. However, the present data do not contain such outliers.

**Table 6:** Descriptive statistics for reaction times in Chinese sentences (*ms*).

Time Expressions	Time Positions	*M*	*SD*
Time Noun	Before subject (TN + S + V + O)	1,623	794
	After subject (S + TN + V + O)	1,486	748
Time Adverb	Before subject (TA + S + V + O)	1,787	1,066
	After subject (S + TA + V + O)	1,334	635

*Note*. *M* = mean. *SD* = standard deviation.

The results of the LME model analysis for the 920 responses are reported in Table 7. While accuracy did not show a significant effect, the trial effect was significant [*t*(851.50) = 3.226, *p* < .01], indicating that as the experiment progressed, participants’ reaction times decreased, suggesting improved task performance. Reaction times were significantly longer when time expressions were positioned before the subject [*t*(43.92) = 5.602, *p* < .001], sugges*t*ing that pre-subject placement of time expressions increases the complexity of syntactic processing, possibly due to a topicalization movement. Additionally, time nouns elicited significantly longer reaction times than time adverbs [*t*(29.76) = 2.397, *p* < .01]. This difference likely arises because time adverbs in *t*he pre-subject position were accepted as correct in only 25% of cases, leading to the exclusion of 75% of trials from the reaction time analysis. Consequently, only highly acceptable sentence stimuli remained, which generally exhibited faster reaction times. Importantly, there was a significant interaction between the position and type of time expression [*t*(30.66) = −3.392, *p* < .01]. This interaction indica*t*ed that the effect of position varies by type: time adverbs caused significantly longer reaction times when placed before the subject, whereas time nouns showed relatively stable reaction times regardless of whether they were positioned before or after the subject.

**Table 7:** LME analysis results for reaction times in Chinese sentences.

Variables	Estimate	*SE*	*df*	*t* value	Pr(>|t|)	*p*
(Intercept)	7.106	0.057	55.22	123.823	*p* < .001	^***^
trial.z	−0.038	0.012	851.50	3.226	*p* < .01	^**^
Position (beforeS)	0.397	0.071	43.92	5.602	*p* < .001	^***^
Type (time noun)	0.120	0.050	29.76	2.397	*p* < .05	^*^
Position^*^Type	−0.283	0.084	30.66	−3.392	*p* < .01	^**^

*Note*. Subjects = 37. Item = 32. Type (time noun and time adverb) = 2. Position (before and after the subject) = 2. Total Observation = 920. *SE* = standard error.

*** *p* < .001.

** *p* < .01.

* *p* < .05.

When examining the positions of time nouns relative to the subject, using the post-subject position (“[Spec, TP]-like” position) as the baseline, it was found that sentences with time nouns placed before the subject were processed significantly more slowly than those with time nouns placed after the subject [*t*(9.530) = 2.476, *p* < .05]. Similarly, analysis of time adverb placement revealed that sentences with time adverbs before the subject were processed significantly more slowly than those with adverbs positioned after the subject [*t*(13.519) = 4.322, *p* < .001]. These findings suggested that pre-subject placement of time expressions increased the complexity of syntactic processing, likely due to a topicalization movement.

## Discussion

Study 3 examined how syntactic position (pre-/post-subject) influences sentence comprehension and cognitive processing of time nouns and time adverbs. Accuracy analyses revealed that time expressions in the pre-subject position were significantly less likely to be judged correct than those in the post-subject position, supporting the hypothesis that the post-subject position (“[Spec, TP]-like” position) serves as the default location for time expressions. Additionally, time nouns and time adverbs exhibited distinct patterns: whereas time nouns maintained relatively stable accuracy in both positions, time adverbs showed significantly lower accuracy when placed before the subject. Notably, the accuracy rate for pre-subject time adverbs was around 25%, emphasizing their stronger reliance on the post-subject position. Reaction time data further reinforced these findings: time expressions positioned before the subject yielded significantly longer reaction times, indicating increased syntactic processing complexity—likely due to topicalization movements.

### General discussion

This study investigated the syntactic positions and characteristics of time nouns and time adverbs in Chinese, examining how these time expressions interact with the language’s grammatical structure. In Study 1, a corpus analysis was conducted to determine the default positions of time nouns and time adverbs relative to the subject in conversational data. Study 2 built on these findings by evaluating the acceptability of time nouns and time adverbs placed before or after the subject, drawing on judgments from native Chinese speakers. Lastly, Study 3 focused on the cognitive foundations of these syntactic preferences, analyzing sentence processing data, including reaction times and accuracy rates, to uncover how these time expressions influence comprehension. Together, the three studies aimed to elucidate the roles and distinctions between time nouns and time adverbs, particularly regarding their syntactic placement. The following discussion addressed these findings in two key dimensions: (1) the distinction between time nouns and time adverbs, and (2) their base position and the role of topicalization.

The findings from the three studies highlighted clear syntactic and functional distinctions between time nouns and time adverbs in Chinese. Although both expressions describe the temporal context of a sentence, they differ fundamentally in how they fulfill this role. Time nouns specify the precise point in time at which an action or event occurs, without altering the verb’s aspect [20,28–36]. They exhibit notable syntactic flexibility, appearing either before or after the subject [15–21]. When placed before the subject, time nouns frame the entire sentence temporally; when placed after the subject, they function as part of the predicate, offering a more specific temporal reference.

In contrast, time adverbs are generally restricted to the post-subject position. Rather than specifying a particular time, they indicate the temporal aspect of the subject’s action [24,27,31,43,54]. Recent research suggests that Mandarin Chinese marks aspect primarily through adverbs [24,54,55]. Because time adverbs grammatically modify verb phrases, they are typically positioned after the subject and before the verb [25,26,36,39,56]. Findings from Study 2 confirmed that time adverbs are most acceptable when placed in this position, reflecting their function as predicate modifiers. Study 3 further underscored that positioning time adverbs before the subject reduces acceptability and slows sentence processing.

However, certain time adverbs, such as 忽然 (*hūrán*, ‘suddenly’) and 随后 (*suíhòu*, ‘subsequently’), deviate from this general pattern, as illustrated in Fig 2, based on the subjective acceptability judgments from Study 2. Under specific contextual or pragmatic conditions, these adverbs can appear before the subject, resembling the behavior of time nouns. According to Yang [25], placing time adverbs in the pre-subject position emphasize the temporal framework that will be elaborated in the subsequent context. For instance, 忽然 often signals an abrupt or unexpected event, preparing the listener or reader for sudden developments. Similarly, when an adverb like 随后 is positioned before the subject, it establishes a temporal link between sentences, connecting sequential events [38]. Zhang [26] further observed that in such cases, these adverbs function as discourse markers, with a phonetic pause strengthening their connective role. This dual function—serving as both temporal markers and discourse connectors—allows certain time adverbs to transcend their usual syntactic constraints, thereby aligning their behavior more closely with that of time nouns in specific circumstances.

The findings of this study strongly supported the hypothesis that the post-subject position (“[Spec, TP]-like” position) serves as the basic placement for time expressions in Chinese. Evidence from all three studies corroborates this conclusion. Study 1 demonstrated that time expressions in natural discourse overwhelmingly favor the post-subject position, Study 2 revealed significantly higher acceptability ratings for time expressions in this position, and Study 3 highlighted the cognitive constraints of the pre-subject position, showing lower accuracy rates and notably longer reaction times, especially for time adverbs.

The positional flexibility of time nouns can be attributed to their capacity for topicalization, which aligns with the topic-comment structure characteristic of Chinese grammar [15,22,23]. For example:

(11) 他第二天早晨就领了水生回去了。

*Tā dìèrtiān zǎochén jiù lǐngle shuǐshēng huíqù le*
‘The next morning, he took Shuisheng back.’

(12) 第二天早晨他就领了水生回去了。

*Dìèrtiān zǎochén tā jiù lǐngle shuǐshēng huíqù le*

(Taken from Wen [20] p. 23)

In Sentence (11), the time nouns 第二天 (*dìèrtiān*, 'the next day') and 早晨 (*zǎochén*, 'morning') appear after the subject, specifying when the action took place. In contrast, in Sentence (12), topicalization shifts the time noun before the subject, emphasizing the time framework. This topicalized structure is a hallmark of standard Chinese usage.

Time adverbs, in contrast, face significant limitations in topicalization because they function as predicate-dependent modifiers. This dependency makes it difficult for them to serve as independent topics. For example:

(13) 他们仍不走。

*Tā men réng bù zǒu.*

‘They’re still not leaving.’

(14) *仍他们不走。

*Réng tā men bù zǒu.*

(Taken from Yang [25], p. 70)

In Sentence (13), the time adverb 仍 (*réng*, 'still') naturally modifies the verb 不走 (*bù zǒu*, 'not leave') when placed after the subject, ensuring smooth syntax, semantic coherence, high acceptability, and faster processing. However, in Sentence (14), positioning 仍 before the subject renders the sentence grammatically incorrect and significantly more difficult to process.

The present study has revealed that Chinese time nouns and time adverbs exhibit clear positional and functional distinctions. Time nouns can appear flexibly before or after the subject, while time adverbs predominantly occupy a post-subject position. These findings confirm that the post-subject position (“[Spec, TP]-like” position) —mirroring tense positions in other languages [8–14]—serves as the basic placement for Chinese time expressions. Moreover, time nouns demonstrate a capacity for topicalization to emphasize the temporal setting, reflecting the topic-comment structure characteristic of Chinese syntax. In contrast, time adverbs, as predicate-dependent modifiers, lack this flexibility.

This preference for positioning time expressions in the post-subject position is further underscored by how Chinese speakers handle time and locative phrases. According to Chao [15], time phrases sound less natural when placed after locative phrases, and Tamaoka & Zhang [57] note that Chinese native speakers often judge time nouns positioned after locative phrases as unnatural or incorrect—unlike Japanese native speakers, who find this order acceptable. These cross-linguistic comparisons highlight both the complexity of Chinese syntax and the robust preference for placing time expressions in “[Spec, TP]-like” position. Taken together, the results of this study shed light on how native Chinese speakers perceive and process time expressions in naturally occurring discourse, offering insights into broader cross-linguistic patterns of temporal reference.

## Future directions

Several avenues for further research emerge from the present findings. First, while this study focused on spoken conversational corpora, future investigations could examine broader text types (e.g., written narratives, academic texts, online forums) to explore whether similar positional preferences and constraints for time expressions persist across different registers. Second, cross-linguistic comparisons, particularly with typologically diverse languages, would shed light on whether the syntactic behaviors of time nouns and time adverbs in Chinese are universal or language-specific. Third, developmental and acquisition studies involving both native-speaking children and second-language learners could provide insights into how and when speakers internalize the notion of the post-subject position (“[Spec, TP]-like” position) as the default for time expressions. Fourth, employing neurolinguistic (e.g., EEG, fMRI) and psycholinguistic (e.g., eye-tracking, ERP) methods could offer more detailed evidence of how the brain processes time expressions, especially when they deviate from the post-subject position. Finally, discourse-pragmatic factors warrant closer attention: examining how speaker intent, information structure, and pragmatic emphasis influence the acceptability and processing of time expressions would deepen our understanding of the subtle interplay between syntax, semantics, and pragmatics in Chinese.

## Supporting information

S1 Appendix Sentences used for Study 2. This appendix lists the sentences used to assess acceptability judgments regarding the placement of time nouns and time adverbs relative to the subject. Sentences are divided into two types: (a) those with the time expression before the subject and (b) those with the time expression after the subject.
(PDF)

S2 Appendix Sentences used for Study 3. This appendix provides the sentences used to analyze reaction times and accuracy rates related to the positioning of time nouns and time adverbs. Sentences are categorized as: (a) those with the time expression preceding the subject and (b) those with the time expression following the subject.
(PDF)

S3 File Data ST1 and ST2. (XLSX)
