# Supplementary material for: Positioning of Chinese time nouns and adverbs: Evidence from corpus, acceptability, and processing studies
Source: PLoS One. 2025 Jul 30;20(7):e0329271. doi: 10.1371/journal.pone.0329271 (PMC12310041; doi:10.1371/journal.pone.0329271)
Supplement: S1 Appendix — This appendix lists the sentences used to assess acceptability judgments regarding the placement of time nouns and time adverbs relative to the subject. Sentences are divided into two types: (a) those with the time expression before the subject and (b) those with the time expression after the subject. (PDF) [file pone.0329271.s001.pdf]

Appendix 1 – Sentences used for study 2

Appendix 1 lists sentences that were utilized to assess acceptability judgments regarding the placement of time nouns and time adverbs relative to the subject in a sentence. The sentences are organized into two main categories: (a) sentences where a time noun or adverb is positioned before the subject, and (b) sentences where a time noun or adverb is positioned after the subject.

Glossary of Terms: NP (Noun Phrase) is a word or group of words that functions in a sentence as subject, object, or prepositional object. V (Verb) indicates the action or state of being in the sentence. ASP (aspect) refers to the aspect marker in Chinese, which indicates the state or temporal quality of a verb. Not every sentence will include an aspect marker.

(1a) TN (Time Noun) + S (Subject) + V (Verb)-aspect + O (Object)

昨天	我	去了	超市。
Zuótiān	*wǒ*	*qù le*	*chāoshì.*
TN(yesterday)	NP-sub(I)	V(go)-ASP	NP-obj(supermarket)

"I went to the supermarket yesterday."

(1b) S (Subject) + TN (Time Noun) + V (Verb)-aspect + O (Object)

我	昨天	去了	超市。
Wǒ	*zuó tiān*	*qù le*	*chāo shì.*
NP-sub(I)	TN(yesterday)	V(go)-ASP	NP-obj(supermarket)

Below are pairs of Chinese sentences with time nouns (a) and (b) accompanied by their English translations.

(2a) 今天我心情很好。 'I feel good today.'

Jīn tiān wǒ xīn qíng hěn hǎo.

(2b) 我今天心情很好。

Wǒ jīn tiān xīn qíng hěn hǎo.

(3a) 明天我们将举行重要会议。 'We will have an important meeting tomorrow.'

Míng tiān wǒ men jiāng jǔ xíng zhòng yào huì yì.

(3b) 我们明天将举行重要会议。

Wǒ men míng tiān jiāng jǔ xíng zhòng yào huì yì.

(4a) 前天我去了新的咖啡馆。'I went to the new cafe the day before yesterday.'

Qián tiān wǒ qù le xīn de kā fēi guǎn.

(4b) 我前天去了新的咖啡馆。

Wǒ qián tiān qù le xīn de kā fēi guǎn.

(5a) 后天我要举办生日派对。I'm having a birthday party the day after tomorrow.

Hòu tiān wǒ yào jǔ bàn shēng rì pài duì.

(5b) 我后天要举办生日派对。

Wǒ hòu tiān yào jǔ bàn shēng rì pài duì.

(6a) 早上我喜欢喝咖啡。I'd like to drink coffee in the morning.

Zǎo shàng wǒ xǐ huān hē kā fēi .

(6b) 我早上喜欢喝咖啡。

Wǒ zǎo shàng xǐ huān hē kā fēi.

(7a) 中午我和同事一起吃午饭。At noon I have lunch with my colleagues.

Zhōng wǔ wǒ hé tóng shì yì qǐ chī wǔ fàn.

(7b) 我和同事中午一起吃午饭。

Wǒ hé tóng shì zhōng wǔ yì qǐ chī wǔ fàn.

(8a) 下午我要去看牙医。I'm going to the dentist this afternoon.

Xià wǔ wǒ yào qù kàn yá yī.

(8b) 我下午要去看牙医。

Wǒ xià wǔ yào qù kàn yá yī.

(9a) 晚上我准备看一部新电影。I'm going to watch a new movie in the evening.

Wǎn shàng wǒ zhǔn bèi kàn yí bù xīn diàn yǐng.

(9b) 我晚上准备看一部新电影。

Wǒ wǎn shàng zhǔn bèi kàn yí bù xīn diàn yǐng.

(10a) 目前我正在做这个项目。I am currently working on this project.

Mù qián wǒ zhèng zài zuò zhè gè xiàng mù.

(10b) 我目前正在做这个项目。

Wǒ mù qián zhèng zài zuò zhè gè xiàng mù.

(11a) 现在我在回答你的问题。Now I'm answering your question.

Xiàn zài wǒ zài huí dá nǐ de wèn tí.

(11b) 我现在在回答你的问题。

Wǒ xiàn zài zài huí dá nǐ de wèn tí.

(12a) 过去我是这个学校的学生。I used to be a student of this school.

Guò qù wǒ shì zhè gè xué xiào de xué shēng.

(12b) 我过去是这个学校的学生。

Wǒ guò qù shì zhè gè xué xiào de xué shēng.

(13a) 将来我要做医生。I want to be a doctor in the future.

Jiāng lái wǒ yào zuò yī sheng.

(13b) 我将来要做医生。

Wǒ jiāng lái yào zuò yī sheng.

(14a) 去年我去了欧洲旅行。I took a trip to Europe last year.

Qù nián wǒ qù le ōu zhōu lǚ xíng.

(14b) 我去年去了欧洲旅行。

Wǒ qù nián qù le ōu zhōu lǚ xíng.

(15a) 今年我要学会游泳。I will learn to swim this year.

Jīn nián wǒ yào xué huì yóu yǒng.

(15b) 我今年要学会游泳。

Wǒ jīn nián yào xué huì yóu yǒng.

Hereafter, the stimulating sentences of time adverbs are listed below.

(16a) TA (Time Adverb) + S (Subject) + V (Verb)-aspect + O (Object)

已经	鹏鹏	完成了	这项任务。
Yǐ jīng	*péng péng*	*wán chéng le*	*zhè xiàng rèn wù.*
TA(already)	NP-sub(Peng Peng)	V(accomplish)-ASP	NP-obj(task)

'Peng Peng has accomplished this task.'

(16b) S (Subject) + TA (Time Adverb) + V (Verb)-aspect + O (Object)

鹏鹏	已经	完成了	这项任务。
Péng péng	*yǐ jīng*	*wán chéng le*	*zhè xiàng rèn wù.*
NP-sub(Peng Peng)	TA(already)	V(accomplish)-ASP	NP-obj(task)

Hereafter, only Chinese sentence pairs and their translations are listed below.

(17a) 就要我去新的公司。'I'm going to a new company.'

Jiù yào wǒ qù xīn de gōng sī.

(17b) 我就要去新的公司。

Wǒ jiù yào qù xīn de gōng sī.

(18a) 正在妈妈学习做饭。'Mother is learning to cook.'

Zhèng zài mā ma xué xí zuò fàn.

(18b) 妈妈正在学习做饭。

Mā ma zhèng zài xué xí zuò fàn.

(19a) 预先领导得知了这个消息。'The leader knew the news in advance.'

Yù xiān lǐng dǎo dé zhī le zhè gè xiāo xī.

(19b) 领导预先得知了这个消息。

Lǐng dǎo yù xiān dé zhī le zhè gè xiāo xī.

(20a) 随后我们前往餐厅用餐。'We then went to the restaurant for dinner.'

Suí hòu wǒ men qián wǎng cān tīng yòng cān.

(20b) 我们随后前往餐厅用餐。

Wǒ men suí hòu qián wǎng cān tīng yòng cān.

(21a) 一同我们去公园。'We go to the park together.'

Yì tóng wǒ men qù gōng yuan.

(21b) 我们一同去公园。

Wǒ men yì tóng qù gōng yuan.

(22a) 逐步小林掌握了绘画技巧。'Xiao Lin gradually mastered the skills of painting.'

Zhú bù xiǎo lín zhǎng wò le huì huà jì qiǎo.

(22b) 小林逐步掌握了绘画技巧。

Xiǎo lín zhú bù zhǎng wò le huì huà jì qiǎo.

(23a) 迟早王明会来的。'Wang Ming will come sooner or later.'

Chí zǎo wáng míng huì lái de.

(23b) 王明迟早会来的。

Wáng míng chí zǎo huì lái de.

(24a) 马上社长要出发去机场。'The president is leaving for the airport soon.'

Mǎ shàng shè zhǎng yào chū fā qù jī chǎng.

(24b) 社长马上要出发去机场。

Shè zhǎng mǎ shàng yào chū fā qù jī chǎng.

(25a) 忽然雨大了起来。'The rain suddenly became heavy.'

Hū rán yǔ dà le qǐ lái 。

(25b) 雨忽然大了起来。

Yǔ hū rán dà le qǐ lái.

(26a) 仍然弟弟不明白。'The younger brother still didn't understand.'

Réng rán dì di bù míng bái.

(26b) 弟弟仍然不明白。

Dì di réng rán bù míng bái.

(27a) 向来丽丽都很害羞。'Lily has always been shy.'

Xiàng lái lì li dōu hěn hài xiū.

(27b) 丽丽向来都很害羞。

Lì li xiàng lái dōu hěn hài xiū.

(28a) 一直班主任在思考这个问题。

'The head teacher has been thinking about this problem.'

Yì zhí bān zhǔ rèn zài sī kǎo zhè gè wèn tí.

(28b) 班主任一直在思考这个问题。

Bān zhǔ rèn yì zhí zài sī kǎo zhè gè wèn tí.

(29a) 总是丁丁迟到。'Ding Ding is always late.'

Zǒng shì dīng dīng chí dào.

(29b) 丁丁总是迟到。

Dīng dīng zǒng shì chí dào.

(30a) 暂且我们停下来休息一下。'Let's stop and rest for a while.'

Zàn qiě wǒ men tíng xià lái xiū xi yí xià.

(30b) 我们暂且停下来休息一下。

Wǒ men zàn qiě tíng xià lái xiū xi yí xià.
