# Supplementary material for: Positioning of Chinese time nouns and adverbs: Evidence from corpus, acceptability, and processing studies
Source: PLoS One. 2025 Jul 30;20(7):e0329271. doi: 10.1371/journal.pone.0329271 (PMC12310041; doi:10.1371/journal.pone.0329271)
Supplement: S2 Appendix — This appendix provides the sentences used to analyze reaction times and accuracy rates related to the positioning of time nouns and time adverbs. Sentences are categorized as: (a) those with the time expression preceding the subject and (b) those with the time expression following the subject. (PDF) [file pone.0329271.s002.pdf]

Appendix 2 – Sentences used for study 3

Appendix 2 lists sentences that were utilized to analyze reaction times and accuracy rates regarding the placement of time nouns and time adverbs relative to the subject in a sentence. The sentences are organized into two main categories: (a) sentences where a time noun or adverb is positioned before the subject, and (b) sentences where a time noun or adverb is positioned after the subject.

Below are pairs of Chinese sentences with time nouns (a) and (b) accompanied by their English translations.

(1a) 昨天子轩完成了作业。‘Zixuan finished his homework yesterday.’

Zuó tiān Zǐxuān wán chéng le zuò yè.

(1b) 子轩昨天完成了作业。

Zǐxuān zuó tiān wán chéng le zuò yè.

(2a) 今天梦琪上了舞蹈课。‘Mengqi attended a dance class today.’

Jīn tiān Mèngqí shàng le wǔ dǎo kè.

(2b) 梦琪今天上了舞蹈课。

Mèngqí jīn tiān shàng le wǔ dǎo kè.

(3a) 明天宇航要去郊游。‘Yuhang will go on an outing tomorrow.’

Míng tiān Yǔháng yào qù jiāo yóu.

(3b) 宇航明天要去郊游。

Yǔháng míng tiān yào qù jiāo yóu.

(4a) 前天梓涵学会了骑自行车。‘Zihan learned to ride a bicycle the day before yesterday.’

Qián tiān Zǐhán xué huì le qí zì xíng chē.

(4b) 梓涵前天学会了骑自行车。

Zǐhán qián tiān xué huì le qí zì xíng chē.

(5a) 后天欣怡要参加演讲比赛。‘Xinyi will participate in a speech competition the day after tomorrow.’

Hòu tiān Xīnyí yào cān jiā yǎn jiǎng bǐ sài.

(5b) 欣怡后天要参加演讲比赛。

Xīnyí hòu tiān yào cān jiā yǎn jiǎng bǐ sài.

(6a) 早上晨晨去了公园。‘Chenchen went to the park in the morning.’

Zǎo shàng Chénchén qù le gōng yuán.

(6b) 晨晨早上去了公园。

Chénchén zǎo shàng qù le gōng yuán.

(7a) 中午思远吃了便当。‘Siyuan had a bento for lunch.’

Zhōng wǔ Sīyuǎn chī le biàn dàng.

(7b) 思远中午吃了便当。

Sīyuǎn zhōng wǔ chī le biàn dàng.

(8a) 下午雨桐要去图书馆。'Yutong will go to the library in the afternoon.'

Xià wǔ Yǔtóng yào qù tú shū guǎn.

(8b) 雨桐下午要去图书馆。

Yǔtóng xià wǔ yào qù tú shū guǎn.

(9a) 晚上泽明要看电影。'Zeming will watch a movie in the evening.'

Wǎn shàng Zémíng yào kàn diàn yǐng.

(9b) 泽明晚上要看电影。

Zémíng wǎn shàng yào kàn diàn yǐng.

(10a) 目前婉儿在准备考试。'Wan'er is currently preparing for an exam.'

Mù qián Wǎn'ér zài zhǔn bèi kǎo shì.

(10b) 婉儿目前在准备考试。

Wǎn'ér mù qián zài zhǔn bèi kǎo shì.

(11a) 现在浩然在写论文。'Haoran is writing a thesis now.'

Xiàn zài Hàorán zài xiě lùn wén.

(11b) 浩然现在在写论文。

Hàorán xiàn zài zài xiě lùn wén.

(12a) 过去妈妈喜欢游泳。'Mom used to enjoy swimming.'

Guò qù māma xǐ huān yóu yǒng.

(12b) 妈妈过去喜欢游泳。

Māma guò qù xǐ huān yóu yǒng.

(13a) 将来王华要出国留学。'Wang Hua will study abroad in the future.'

Jiāng lái Wáng Huá yào chū guó liú xué.

(13b) 王华将来要出国留学。

Wáng Huá jiāng lái yào chū guó liú xué.

(14a) 去年张薇参加了马拉松。'Zhang Wei participated in a marathon last year.'

Qù nián Zhāng Wēi cān jiā le mǎ lā sōng.

(14b) 张薇去年参加了马拉松。

Zhāng Wēi qù nián cān jiā le mǎ lā sōng.

(15a) 今年若琳考上了大学。'Ruolin got into college this year.'

Jīn nián Ruòlín kǎo shàng le dà xué.

(15b) 若琳今年考上了大学。

Ruòlín jīn nián kǎo shàng le dà xué.

(16a) 明年丽华要参加高考。'Lihua will take the college entrance exam next year.'

Míng nián Lìhuá yào cān jiā gāo kǎo.

(16b) 丽华明年要参加高考。

Lìhuá míng nián yào cān jiā gāo kǎo.

Hereafter, the stimulating sentences of time adverbs are listed below.

(17a) 已经张伟完成了任务。'Zhang Wei has already completed the task.'

Yǐ jīng Zhāng Wěi wán chéng le rèn wù.

(17b) 张伟已经完成了任务。

Zhāng Wěi yǐ jīng wán chéng le rèn wù.

(18a) 预先李婷准备好了材料。'Liting prepared the materials in advance.'

Yù xiān Lǐtíng zhǔn bèi hǎo le cái liào.

(18b) 李婷预先准备好了材料。

Lǐtíng yù xiān zhǔn bèi hǎo le cái liào.

(19a) 随后大家开始讨论。'Everyone started the discussion afterward.'

Suí hòu dà jiā kāi shǐ tǎo lùn.

(19b) 大家随后开始讨论。

Dà jiā suí hòu kāi shǐ tǎo lùn.

(20a) 逐步赵刚解决问题。'Zhao Gang solved the problem step by step.'

Zhú bù Zhào Gāng jiě jué wèn tí.

(20b) 赵刚逐步解决问题。

Zhào Gāng zhú bù jiě jué wèn tí.

(21a) 马上刘梅要出发了。'Liu Mei is about to set off.'

Mǎ shàng Liú Méi yào chū fā le.

(21b) 刘梅马上要出发了。

Liú Méi mǎ shàng yào chū fā le.

(22a) 忽然李明发现了问题。'Li Ming suddenly discovered a problem.'

Hū rán Lǐ Míng fā xiàn le wèn tí.

(22b) 李明忽然发现了问题。

Lǐ Míng hū rán fā xiàn le wèn tí.

(23a) 仍然陈明坚持自己的观点。'Chen Ming still insists on his opinion.'

Réng rán Chén Míng jiān chí zì jǐ de guān diǎn.

(23b) 陈明仍然坚持自己的观点。

Chén Míng réng rán jiān chí zì jǐ de guān diǎn.

(24a) 一直孙总支持这个项目。'General Sun has always supported this project.'

Yī zhí Sūn Zǒng zhī chí zhè gè xiàng mù.

(24b) 孙总一直支持这个项目。

Sūn Zǒng yī zhí zhī chí zhè gè xiàng mù.

(25a) 总是周华帮助别人。'Zhou Hua always helps others.'

Zǒng shì Zhōu Huá bāng zhù bié rén.

(25b) 周华总是帮助别人。

Zhōu Huá zǒng shì bāng zhù bié rén.

(26a) 暂且张强放下工作。'Zhang Qiang temporarily set aside his work.'

Zàn qiě Zhāng Qiáng fàng xià gōng zuò.

(26b) 张强暂且放下工作。

Zhāng Qiáng zàn qiě fàng xià gōng zuò.

(27a) 就要李婷去机场。'Liting is about to go to the airport.'

Jiù yào Lǐ Tíng qù jī chǎng.

(27b) 李婷就要去机场。

Lǐ Tíng jiù yào qù jī chǎng.

(28a) 正在郭涛审阅论文。'Guo Tao is reviewing the thesis.'

Zhèng zài Guō Tāo shěn yuè lùn wén.

(28b) 郭涛正在审阅论文。

Guō Tāo zhèng zài shěn yuè lùn wén.

(29a) 一同李明和王刚去图书馆。'Li Ming and Wang Gang went to the library together.'

Yī tóng Lǐ Míng hé Wáng Gāng qù tú shū guǎn.

(29b) 李明和王刚一同去图书馆。

Lǐ Míng hé Wáng Gāng yī tóng qù tú shū guǎn.

(30a) 迟早工程师会找到解决方案。'The engineer will eventually find a solution.'

Chí zǎo gōng chéng shī huì zhǎo dào jiě jué fāng àn.

(30b) 工程师迟早会找到解决方案。

Gōng chéng shī chí zǎo huì zhǎo dào jiě jué fāng àn.

(31a) 向来爸爸都很守时。'Dad has always been very punctual.'

Xiàng lái bàba dōu hěn shǒu shí.

(31b) 爸爸向来都很守时。

Bàba xiàng lái dōu hěn shǒu shí.

(32a) 永远小李支持小王。'Xiao Li will always support Xiao Wang.'

Yǒng yuǎn Xiǎo Lǐ zhī chí Xiǎo Wáng.

(32b) 小李永远支持小王。

Xiǎo Lǐ yǒng yuǎn zhī chí Xiǎo Wáng.
